# Going “Up” to Move Forward: S-1 Bifactor Models and the Study of Neurocognitive Abilities in Psychopathology

**DOI:** 10.3390/ijerph18147413

**Published:** 2021-07-11

**Authors:** Darren Haywood, Frank D. Baughman, Barbara A. Mullan, Karen R. Heslop

**Affiliations:** 1 Discipline of Psychology, School of Population Health, Curtin University, Perth 6845, Australia; frank.baughman@curtin.edu.au (F.D.B.); barbara.mullan@curtin.edu.au (B.A.M.); 2 Health Psychology & Behavioural Medicine Research Group, School of Population Health, Curtin University, Perth 6845, Australia; 3 WA Cancer Prevention Research Unit, School of Population Health, Curtin University, Perth 6845, Australia; 4 Curtin School of Nursing, Curtin University, Perth 6845, Australia; k.heslop@curtin.edu.au

**Keywords:** S-1 bifactor, bifactor, S-1, neurocognition, executive functioning, intelligence, IQ, psychopathology, *p*-factor, CFA, simulation

## Abstract

Recently, structural models of psychopathology, that address the diagnostic stability and comorbidity issues of the traditional nosological approach, have dominated much of the psychopathology literature. Structural approaches have given rise to the *p*-factor, which is claimed to reflect an individual’s propensity toward all common psychopathological symptoms. Neurocognitive abilities are argued to be important to the development and maintenance of a wide range of disorders, and have been suggested as an important driver of the *p*-factor. However, recent evidence argues against *p* being an interpretable substantive construct, limiting conclusions that can be drawn from associations between *p*, the specific factors of a psychopathology model, and neurocognitive abilities. Here, we argue for the use of the S-1 bifactor approach, where the general factor is defined by neurocognitive abilities, to explore the association between neurocognitive performance and a wide range of psychopathological symptoms. We use simulation techniques to give examples of how S-1 bifactor models can be used to examine this relationship, and how the results can be interpreted.

## 1. Introduction

In recent years, there has been a body of research that has moved away from the study of any single disorder (see [1]). This work is driven by issues of high comorbidity and low diagnostic stability within the traditional nosological approach to diagnosis (see [2,3,4]). In particular, the rise of dimensional structural models of psychopathology has led to explorations of the commonalities of disorders, as well as what may underpin these commonalities [1]. One of the most prominent structural models of psychopathology, the bifactor model, revealed that a significant amount of variance from symptoms of a range of disorders could be accounted for by a single general factor at the “top” of the model, while specific disorder variance could be largely accounted for by a group of lower-order, or “specific” factors, such as externalising, internalising and thought disorder (e.g., [5]). The general factor was termed the *p*-factor, likened to the *g* factor of intelligence, and said to be a normally distributed property across the population that determines an individual’s propensity toward all common psychopathological symptoms [5]. A range of research, with little consensus, has attempted to uncover what is the substantive construct of *p*, or in other words, what *p* represents. For example, the *p*-factor has been claimed to reflect neuroticism [6], disordered thought [7], functional impairment [8], and impulsive responsivity to emotion [9]. Furthermore, constructs, such as neurocognitive abilities, due to their reliable correlation with the general factor, have been claimed to be a key driver of the *p*-factor [1].

Higher-order neurocognitive abilities have long been theorized to be important components and processes underlying the development and maintenance of psychopathology (e.g., [10,11,12,13]). However, evidence of the contributions of higher-order neurocognitive abilities to psychopathology is often inconsistent (e.g., [14,15,16,17]). One possible reason for this heterogeneity may be the diagnostic instability and comorbidity present in research grounded in the nosological approach (e.g., see [13]). Therefore, exploring how neurocognitive abilities may contribute to *p*, and the specific factors of psychopathology using the dimensional based structural approach, is appealing.

However, recently, there has been strong evidence against *p* as a substantive construct. Murray et al. [18] and Snyder and Hankin [19] explain that the *p*-factor, is a function of the sample from which it is derived. Levin-Aspenson, et al. [20] demonstrated that the *p*-factor derived from two different samples is a substantially different construct, and Fried, et al. [21] showed that, statistically, *p* is simply a representation of the combination of an individual’s diagnosis. Furthermore, using simulation methodologies, Greene, et al. [22] showed that fit indices, often used to champion the bifactor model (with a *p*-factor) over a correlated factors model (without a *p*-factor), unfairly bias the more accommodating bifactor model. Correlations between specific factors in a bifactor model also often switch signs when compared to the specific factor associations in the correlated factors models (e.g., [5]), and these changes do not have a strong theoretical explanation [23]. Furthermore, recently, we have demonstrated the particular lack of applicability and consistency of the *p*-factor within subgroups of a population [24], limiting the possibility of a universal substantive *p*. Ultimately, Lahey et al. [1] explains that *p* is simply “…a “weighted average” of some aspects of all symptoms exhibited by each person at that point in time” (p. 61), and it is unclear whether *p* can have any substantive, theoretical meaning.

As neurocognitive abilities are associated with a wide range of disorders, *p* as a substantive construct has promise for increasing our understanding of how neurocognitive abilities are involved in the development, maintenance, and treatment of psychopathology. However, without a theoretical consensus on what *p* is (See [20,21,25]), it may not greatly enhance our understanding of the association between neurocognitive abilities and psychopathology. Relatedly, as *p* is inherently fluid, changes in the makeup of *p* also result in substantive changes to the specific factors of the models, further limiting our ability to consistently interpret the associations between neurocognitive abilities and psychopathology. To combat the statistical concerns of *p*, alternative bifactor models have been developed. In particular, there has been increasing interest in the use of the S-1 bifactor model in the study of psychopathology [26,27,28]. The S-1 bifactor model includes a “reference domain” that acts to predefine the meaning of the general factor (see [26,27,28] for detailed explainations). The predefining of the general factor removes the issues presented in the traditional bifactor literature in that *p* is an undefined, flexible statistical construct [28]. An S-1 bifactor model includes a reference domain with typically two or more indicators that load only onto the general factor, while the other indicators load onto the general factor as well as a specific factor [27]. The general factor in a S-1 bifactor model therefore represents the reference domain, and the specific factors represent the “…true score variance in non-reference symptom facets that is not shared with the general reference factor” ([27], p. 885). Further, the correlations between the specific factors represent the shared variance between the two factors that is not common with the general factor. Heinrich et al. [26] explain that when Caspi et al. [5] removed the thought disorder factor from their bifactor model, due to a Heywood case (an indicator with negative variance), their model became an S-1 bifactor model and the thought disorder factor became the reference domain for the *p*. Effectively, *p* in Caspi et al.’s [5] revised bifactor model *became* the thought disorder factor, rather than a general factor of psychopathology [26]. This demonstrates the difficulties with an undefined general factor (e.g., the *p*-factor), because as *p* is a non-stable statistical weighted summary of symptoms, it is susceptible to changes in meaning in line with changes in model structure and indicators. Therefore, currently, knowledge stemming from associations between the *p*-factor and theoretically important constructs and processes, such as neurocognitive abilities, lack substantive meaning. In contrast, S-1 bifactor models allow us to predefine the meaning of the general factor with a theoretically outstanding candidate [28] and, as the general factor has substantive meaning, unexpected or novel findings, such as specific factors switching signs, could have clear theoretical interpretations and facilitate hypotheses development. Furthermore, a large limitation of traditional bifactor models is inconsistency. However, the inclusion of a reference domain means that the S-1 bifactor models are consistent and therefore replicable [28]. Previously, S-1 bifactor models have predominantly been used with a symptom domain as the general factor. However, as Greene et al. [29] states, any such etiological domain of interest could be modelled as the general factor in an S-1 bifactor model and facilitate the exploration of that domain and psychopathology.

Recently, we called for the use of S-1 bifactor models in the study of associations between neurocognitive abilities and psychopathology, by using neurocognitive abilities as the reference domain [24,30]. S-1 bifactor models account for many of the limitations of both the traditional nosological approach, and the traditional bifactor models. In the following sections, we use data simulation methods to illustrate how S-1 bifactor models could be used to examine the association between neurocognitive abilities and psychopathology and how, even when a S-1 bifactor model has unexpected results, it is able to facilitate a theoretical interpretation and hypotheses development.

## 2. Methods and Analyses

### 2.1. Data Generation

#### 2.1.1. Symptoms

Caspi et al. [5] developed and tested models of psychopathology using data from the Dunedin Multidisciplinary Health and Development Study (total *N* = 1037, *N* = 1000 used by Caspi et al. [5]). The symptom data were gathered using the Diagnostic Interview Schedule [31] and comprised of the number of DSM-IV symptoms with which each individual presented for a range of common disorders (see [5]). Caspi et al. [5] further used a range of potential correlates of psychopathological symptoms from the Dunedin Multidisciplinary Health and Development Study, including measures of neurocognitive ability, to further examine their models. To develop our simulated data, we used a top-down approach from previous work (see [24]) that is similar to Greene et al.’s [22] approach in order to develop a data set comprising of 11 disorder variables for 10,000 participants. Specifically, we used the loadings of Caspi et al.’s [5] revised bifactor model to develop 11 continuous variables representing the symptom counts of (1) alcohol use, (2) cannabis use, (3) hard drug use, (4) tobacco use, (5) conduct disorder, (6) fears and phobias, (7) major depressive episode, (8) generalised anxiety disorder, (9) obsessive compulsive disorder, (10) mania, and (11) schizophrenia, respectively. Like Greene et al. [22], we then assigned a skew of approximately positive skew of 2.0 across the variables to represent the distributions of symptoms typically found in the general population [32]. All data generation and analysis were conducted with RStudio using the Lavaan package [33].

#### 2.1.2. Intelligence

Following the development of the symptom counts, we fitted the Caspi et al. [5] revised bifactor model to the data (details are presented in the analysis section) and saved the factor loadings back to the data set. We then developed three variables simulating Caspi et al.’s [5] measures of the intelligence quotient (IQ) from (1) the Stanford–Binet Intelligence Scale (age 5), (2) the Wechsler Intelligence Scale for Children-Revised (WISC-R; ages 7–11), and (3) the Wechsler Adult Intelligence Scale-IV (WAIS-IV) full scale. The data for the three IQ measures were based on the correlations between each measure of IQ and externalising, internalising and the *p*-factor from Caspi et al.’s [5] revised bifactor model, as well as the correlations between each of the IQ measures from longitudinal research [34,35]. The IQ variables were normally distributed and standardised to a mean of 100 and a standard deviation of 15.

#### 2.1.3. Executive Functioning

We also developed, and added to the data set, variables representing two of Caspi et al.’s [5] measures of executive functioning, the Trail Making Test-B (TMT-B) and the Cambridge Neuropsychological Test Automated Battery—Rapid Visual Information Processing task (CANTAB: RVIP). We developed these data based on the correlations between the two executive functioning measures and externalising, internalising and the *p*-factor from Caspi et al.’s [5] revised bifactor model, as well as correlations found within the literature between the two executive functioning variables, and adult measures of IQ [36,37,38]. To illustrate how S-1 bifactor models can facilitate the interpretation of novel findings, we also developed a second set of data for the TMT-B and the CANTAB: RIVP. We developed the second set of data for these measures based on the unlikely scenario of the measures having a *r* = 0.8 correlation, an undefined association with *g*, no correlation with externalising or internalising, but a maximum possible association with *p* from the revised bifactor model (actual correlations differed slightly to the assigned correlations in the data producing code, due to random data generation factors and association compatibility constraints).

### 2.2. Analyses

**Figure 1:**
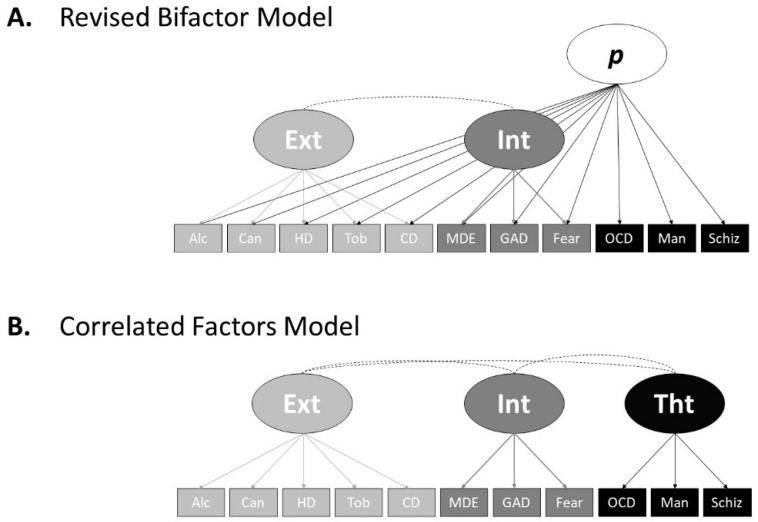
Caspi et al.’s [5] Confirmatory Factor Analysis Models. (**A**) Revised Bifactor Model. (**B**) Correlated Factors Model. Alc = Alcohol. Can = Cannabis. HD = Hard Drugs. Tob = Tobacco, CD = Conduct Disorder. MDE = Major Depressive Episode. GAD = Generalized Anxiety Disorder. Fear = Fears and Phobias. OCD = Obsessive Compulsive Disorder. Man = Mania. Schiz = Schizophrenia. Ext = Externalising. Int = Internalising. Tht = Thought Disorder.

First, to validate the simulated data set, and to act as a comparison to the S-1 bifactor models, we tested the fit of two of Caspi et al.’s [5] structural models (see Figure 1), (A) the revised bifactor model that the simulated data was based on, and (B) the correlated factors model, a popular model in psychopathology research (e.g., see [5]).

For both confirmatory factor analyses (CFAs), we used a maximum likelihood estimation with robust standard errors (MLR), and Pearson’s correlations in RStudio. MLR is robust to deviations of normality, such as symptom count data, by correcting chi-square statistics and standard errors to compensate for skewed data. MLR is also widely used in psychopathology research (see [22]) and is used for continuous indicators, such as our symptom count dimensions. We used the root mean square error of approximation (RMSEA; [39]), the Tucker–Lewis Index (TLI) and the comparative fit index (CFI; [40]) to determine model fit, while we also report standardised root mean square residual (SRMR; [41]). A good-fitting model was determined by RMSEA values of <0.05 [42] and CFI and TLI values of >0.95. However, it is important to note that good model fit, is not a theoretically robust way to choose a model and is not the focus of this research (e.g., see [22]). Rather, the loadings patterns and specific factor covariance will be the focus in this paper.

**Figure 2:**
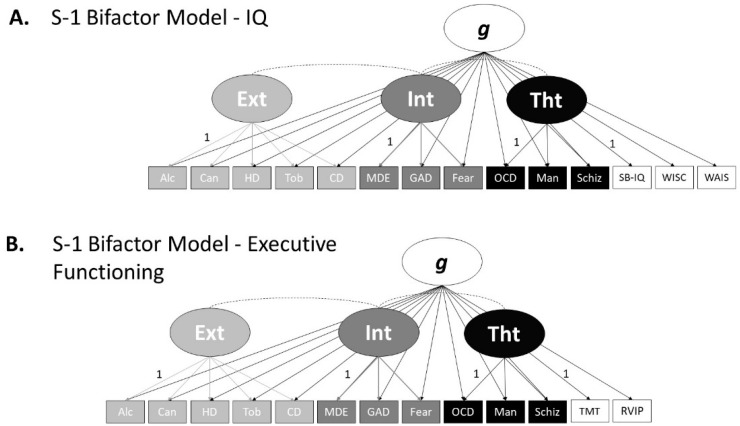
S-1 Bifactor Confirmatory Factor Analysis Models. (**A**) S-1 Bifactor Model—IQ. (**B**) S-1 Bifactor Model Executive Functioning. IQ = Intelligence Quotient. Alc = Alcohol. Can = Cannabis. HD = Hard Drugs. Tob = Tobacco, CD = Conduct Disorder. MDE = Major Depressive Episode. GAD = Generalized Anxiety Disorder. Fear = Fears and Phobias. OCD = Obsessive Compulsive Disorder. Man = Mania, Schiz = Schizophrenia. SB-IQ = Stanford–Binet Intelligence Scale Intelligence Quotient. WISC = Wechsler Intelligence Scale for Children—Intelligence Quotient. WAIS = Wechsler Adult Intelligence—Intelligence Quotient. TMT = Trail Making Test. RVIP = Rapid Visual Information Processing. Ext = Externalising. Int = Internalising. Tht = Thought Disorder. *g* = General Factor.

Next, we tested the fit of three S-1 bifactor models using the simulated data sets (see Figure 2). The first S-1 bifactor model (Figure 2A) used IQ over time, measured by (1) the Stanford–Binet Intelligence Scale (age 5), (2) the WISC-R (ages 7–11), and (3) the WAIS-IV full scale, as the reference domain for the general factor (in this model, the IQ factor), and externalising, internalising and thought disorder as specific factors. Each disorder loaded onto the general factor as well as one of the specific factors as per Caspi et al. [5]. Following the directions of Burns [27], Heinrich et al. [26], and Eid [28], the unstandardised loading of the first indicator of the reference factor, and each specific factor, was fixed to 1, and acted as a reference indicator for that factor. The fit of the S-1 bifactor models was determined using the same criteria as the revised bifactor and correlated factors models.

The second S-1 bifactor model (see Figure 2B) used the same specifications as above. However, executive functioning, measured by the TMT-B and the CANTAB: RVIP, acted as the reference domain for the general factor (in this model, the executive functioning factor) and the first indicator of the factor (TMT-B) acted as the reference indicator. Externalising, internalising and thought disorder remained the specific factors and their first indicator, respectively, remained as the reference indicator.

The final S-1 bifactor model was used to illustrate that even when unexpected, or novel findings occur in S-1 bifactor models, they may have theoretical explanations and drive hypotheses. The S-1 bifactor model was identical to the executive functioning reference domain model above (Figure 2B). However, unlike the model above, which used simulated executive functioning data based on empirical research, this model used data for the TMT-B and CANTAB: RVIP, based on a very unlikely combination of correlations (see data generation section).

## 3. Results

The simulated data fit the Caspi et al. [5] revised bifactor model (Figure 1A) well, (χ2(35, *N* = 10,000) = 44.78, CFI = 1.00, TLI = 1.00, SRMR = 0.005, RMSEA = 0.004, 90% confidence interval (CI) = [0.000, 0.009]), as well as the correlated factors model (Figure 1B), (χ2(41, *N* = 10,000) = 432.27, CFI = 0.990, TLI = 0.987, SRMR = 0.29, RMSEA = 0.031, 90% CI = [0.028, 0.034]). Table 1 shows the loadings and association characteristics of the revised bifactor model, and Table 2 shows the loadings and association characteristics for the correlated factors model. The factor loadings and correlations with IQ and executive functioning do slightly differ to Caspi et al. [5] due to random data generation factors, potential skew differences between Caspi et al.’s [5] data and the simulation data, correlation compatibility constrains, and the use of continuous instead of ordinal variables. However, our models’ loading patterns and characteristics, as well as factor associations with IQ and executive functioning, closely resemble that of Caspi et al. [5]. Therefore, we conclude that our simulated data represent that of Caspi et al. [5] well.

Table 3 shows the loadings and association characteristics of the first S-1 bifactor model (Figure 2A). This S-1 bifactor model used IQ over time as the reference domain and the first indicator of each factor as the reference indicator. The data fit the model well (χ2(63, *N* = 10,000) = 787.91, CFI = 0.988 TLI = 0.982, SRMR = 0.030, RMSEA = 0.034, 90% CI = [0.032, 0.036]). Largely, as the IQ general factor’s loadings on the symptom indicators show, the IQ general factor did poorly at accounting for variance amongst the symptoms. However, on closer inspection, the IQ general factor accounted for notably more variance amongst the internalising and thought disorder indicators when compared to the externalising indicators. This mirrors the trend of bivariate correlations between the specific factors and the measures of IQ in the correlated factors model above. In the correlated factors model, associations between the specific factors and the measures of IQ represent the correlations between the variance of the items that load onto each specific factor, respectively. In the S-1 bifactor model, loadings of indicators on the general, predefined, factor represent the variance of each indicator that is accounted for by that factor. The S-1 bifactor model therefore not only answers a different research question (e.g., “what amount of variance of each symptom can be accounted for by the general predefined factor?”) when compared the correlated factors model, but also allows us to explore the partial associations between the specific factors after accounting for the general predefined factor. As Table 3 shows, the covariation between the specific factors fell slightly when compared to the correlated factors model (Table 2) and fell by a similar magnitude, indicating that the IQ general factor accounts for a small amount of the association between externalising, internalising and thought disorder.

Table 4 shows the loadings and association characteristics of the second S-1 bifactor model (Figure 2B). This S-1 bifactor model used executive functioning as the reference domain and the first indicator of each factor as the reference indicator. The data fit the model well (χ2(51, *N* = 10,000) = 419.96, CFI = 0.991, TLI = 0.987, SRMR = 0.023, RMSEA = 0.027, 90% CI = [0.025, 0.029]). The loading of the CANTAB-RVIP was negative as lower TMT-B scores reflect better performance and TMT-B was the reference indicator for the general factor. The executive functioning referenced general factor did better than the IQ referenced general factor in accounting for variance amongst the symptoms. However, the executive functioning general factor accounted for notably less variance amongst the first three externalising indicators (alcohol, cannabis, and hard drugs use) when compared to the rest of the symptoms. The executive functioning general factor did better than the IQ general factor at accounting for tobacco use and conduct disorder symptoms, comparatively similar when accounting for internalising indicators, and better when accounting for thought disorder indicators. The finding that the executive functioning general factor did notably poorer at accounting for alcohol, cannabis and hard drugs use when compared to the other indicators might inform hypotheses regarding their aetiological interrelations and separability when compared to other symptomology. As Table 3 shows, like with the IQ referenced S-1 model, partial covariation between the specific factors fell slightly when compared to the correlated factors model (Table 2). However, association between specific factors fell by a greater magnitude when compared to the IQ referenced domain S-1 model. The association between the externalising and thought disorder factors, and the association between internalising and thought disorder factors fell, when compared to the correlated factors model by a similar magnitude (0.18 and 0.16, respectively). However, the association between externalising and internalising fell to a greater extent (0.34). This may inform hypotheses, such as executive functioning mediating the association between internalising symptoms (e.g., generalised anxiety) and externalising behaviours (e.g., substance use).

Lastly, to demonstrate how S-1 bifactor models facilitate hypothesis generation from unexpected or novel findings, we tested the same S-1 bifactor model above, but with executive functioning data with a highly unexpected correlation matrix (see Data Generation section). The data fit the model well (χ2(51, *N* = 10,000) = 215.17, CFI = 0.997, TLI = 0.996, SRMR = 0.12, RMSEA = 0.018, 90% CI = [0.18, 0.020]). The loadings and associations of this model are displayed in Table 5. The general factor, with executive functioning as the reference domain, did well in accounting for variance amongst the symptoms. As expected, due to the measures’ assigned correlation with *p* from the revised bifactor model, the thought disorder indicators loaded the highest on the executive functioning general factor in this S-1 bifactor model. Loadings of the externalising and internalising indicators on the executive functioning general factor were also comparatively high, due to the common variance between the specific factors, even although they were assigned not to correlate in the revised bifactor model during data generation. Due to the highly abnormal executive functioning data, partial associations between the specific factors differ extensively when compared to the correlated factors model and the previous S-1 bifactors models. The covariation between the externalising and internalising factors switched signs, and the factors had very little association (−0.044), while the covariation of the externalising and thought disorder factors, and the internalising and thought disorder factors, dropped substantially when compared to the correlated factors model (0.115 and 0.191). If we were to interpret these patterns of covariation, in particular the negative externalising and internalising factors association, in a standard bifactor model there would be little theoretical reason for the association to change signs, and substantive interpretation would be difficult due to the ambiguity of the *p*-factor (see [23]). However, in a S-1 bifactor model the general factor is defined *a*-priori. The knowledge of what the general factor represents, in this case executive functioning, allows us to make substantive interpretations of the changes in specific factor associations and develop hypotheses as a result. For example, the substantial reduction in the association between the externalising and internalising factors in this S-1 bifactor model, when compared to the correlated factors model, may have led to the hypothesis that executive functioning is a full mediator of the association between internalising symptoms, such as depression or anxiety, and externalising behaviours, such as substance use. This demonstrates the utility of using of a typical structural model, the correlated factors model, in conjunction with the S-1 bifactor model for data interpretation. Furthermore, these results may have suggested that, when executive functioning is accounted for, those with more internalising symptoms are conversely slightly less inclined to externalising behaviours. This hypothetical illustration shows that theory building, and testing is a useful characteristic of S-1 bifactor models.

## 4. Discussion

In this paper, we provided the case for the use of S-1 bifactor models in the exploration of neurocognitive abilities in psychopathology. We used simulation methodologies to show how no matter the results of a S-1 bifactor model, using neurocognitive abilities as a reference domain, due to the general factor reflecting a substantive construct, an interpretable hypothesis or theoretical explanation could emerge. S-1 bifactor models account for the issues of substantive and statistical inconsistency of an undefined general factor in psychopathology research [26,27,28]. We provided three examples of how S-1 bifactor models could be used to further our understanding of the associations between neurocognitive abilities and psychopathology. In our first example, we used IQ over time as the reference domain for our general factor with externalising, internalising and thought disorder serving as the specific factors. The IQ general factor accounted for a small amount of variance among the symptoms, with the thought disorder indicators generally having the strongest loading on the IQ general factor. This example showed the utility of the S-1 bifactor approach over solely the the correlated factors model. Using the S-1 approach we could see the loading of each specific disorder on the IQ general factor, allowing us to examine the proportion of variance in each indicator that was accounted for by the IQ general factor, as well as the indicators loadings on the specific factors. For example, if we look to the associations between the measures of IQ and the externalising, internalising and thought disorder factors in the correlated factors model, we see generally consistent strengths of association (minus WISC-IQ and externalising). However, this only tells part of the story, as the factors reflect the common variance amongst their specific indicators, and not the common variance amongst the indicators after the general factor has been taken into account, as per the S-1 model. Therefore, while correlated factors models can show us the association between IQ and the common variance of indicators for each specific factor, the S-1 bifactor model can show us the common variance amongst the specific indicators once IQ has been taken into account, as well as the loadings of each specific indicator on IQ. Therefore, given the attributes of each approach, we suggest that the correlated factors model and the S-1 bifactor model should be used in parallel to answer different research questions and provide a range of evidence assessing the association between neurocognitive abilities and psychopathology.

Our second example used executive functioning as the reference domain for the general factor. In this example, the executive functioning general factor typically accounted for more variance in psychopathology symptoms when compared to the IQ general factor. The externalising indicators had the lowest loadings on the executive functioning general factor when compared to the internalising or thought disorder indicators. However, as S-1 bifactor models allow us to examine the loadings of each indicator on the predefined general factor, we can see that alcohol and cannabis use had noticeably lower loadings on the executive functioning general factor when compared to the other indicators. This indicates that, in this instance, when compared to other disorders/symptoms, executive functioning did not have as much utility in accounting for alcohol and cannabis use. Results such as this, due to the knowledge of what the substantive construct of the general factor is, can drive hypotheses for future work. In this model, the associations between the specific factors all fell when compared to the correlated factors model. In particular, the associations between the externalising and internalising factors fell notably. As in S-1 bifactor models the associations between the specific factors are partial associations after accounting for the predefined general factor, it may be possible to hypothesise that executive functioning may be particularly important in the association between internalising symptoms (e.g., anxiety) and externalising behaviours (e.g., substance use).

Lastly, we used the same symptom data but developed very unlikely data for the two measures of executive functioning. In the data simulation code, the two measures were made to be highly correlated with each other and the *p*-factor from the revised bifactor model, but that had almost no correlation with the externalising and internalising factors from that model. These data were developed to illustrate that even when models go “wrong” when using a S-1 bifactor approach, the results still have substantive interpretation due to the known “meaning” of the general factor. In this S-1 model, the general factor, referenced by executive functioning, did well in accounting for symptomology. Loadings on the executive functioning general factor were typically high when compared to the previous models and the thought disorder indicators again had the strongest loadings on the executive functioning general factor. Here, our focus is on the covariation between the specific factors externalising, internalising and thought disorder. Traditional bifactor models often result in the associations between the specific factors changing signs and differing substantially from the correlated factors model (e.g., [5]). Pettersson et al. [23] points out that due to the unspecified nature of the *p*-factor in a traditional bifactor model, associations between the specific factors changing substantially are difficult to interpret and have no clear theoretical explanation. However, if this occurs in a S-1 bifactor model, due to the a priori specification of the general factor, if the associations between specific factors do change substantially, it can be clearly interpreted. In our example, the association between externalising and internalising fell substantially and the two factors were negatively associated. As we clearly understand what the associations between the specific factors in a S-1 bifactor model represent, we may use the results to develop hypotheses. In our example, we may, for instance, hypothesise that executive functioning is all important in the association between internalising symptoms and externalising behaviours, such that executive functioning is a full mediator. Then, as the general factor is predefined, further research could attempt to replicate and build upon this finding. This is impossible using standard bifactor approaches. This research was conducted to inform the use of the S-1 bifactor approach for the study of neurocognitive abilities in psychopathology in the research setting. However, recently, there have been suggestions that structural models such as these may inform a clinicians’ practices in the treatment setting. A detailed discussion regarding the utility of these approaches in a treatment setting is beyond the scope of this research. However, Ruggero et al. [43] provides information and a case illustration of how structural approaches can guide clinical practice. For example, a clinician taking a dimensional structural approach might, instead of viewing a clients’ symptoms as representing a certain diagnosis, view the symptoms as dimensional indicators that share commonality and relations at different hierarchical levels (see [43] for a detailed demonstration). With regard to the clinical usefulness of the exploration of neurocognitive abilities using the S-1 bifactor approach, it is possible to use the patterns of loadings typically presented between the general neurocognitive factor and a range of symptomology to inform how the specific collection of symptoms (and their severity) an individual client is experiencing may be functionally associated with their neurocognitive performance, and direct their treatment accordingly.

Limitations of the S-1 Bifactor Approach in the Study of Neurocognitive Abilities and Psychopathology

It is important to acknowledge the potential limitations of the S-1 bifactor approach for studying neurocognitive abilities in psychopathology. There are three primary limitations for using the S-1 bifactor approach: first, the sole use of the S-1 approach would remove the ability to examine the associations of particular neurocognitive abilities and psychopathological symptoms or factors. However, the combined use of the S-1 approach and the correlated factors model (with bivariate correlations) mitigates this issue. Second, similar to other structural models (see [24]), it is likely that, the S-1 bifactor model would have limited utility subgroups of a population. However, as the general factor is predefined S-1 bifactor models would likely have better consistency when compared to other structural models. This means that the S-1 bifactor model may only be useful for a general population sample, or a sample with large variability in symptoms and limited symptom heterogeneity (see [24]). Third, given that having neurocognitive abilities modelled as the general factor results in symptoms loading directly on the factor, it limits our ability to explore nuanced proposals (i.e., the multidimensional hypothesis [44]) of the heterogeneity of neurocognition within psychopathology on the individual level. However, again mitigating this issue by using the S-1 approach in conjunction with the correlated factors model approach, it may be possible to explore the heterogeneity of neurocognitive abilities within the factors derived from a correlated factors model on the individual level [30].

## 5. Limitations of the Research and Directions for Future Research

This study, while demonstrating the usefulness of the S-1 bifactor model to explore neurocognitive abilities and psychopathology, did have some limitations. First, all data used were simulated from Caspi et al. [5]. The simulated data approach allowed for useful control over the data to facilitate the demonstration of different modelling circumstances (e.g., the data based on a very unlikely combination of executive functioning correlations). However, the results should be considered as a demonstration of the use of the S-1 bifactor model in this context, rather than used to elucidate any important contributions of neurocognitive abilities toward psychopathology. In line with this, we developed continuous data for 11 disorder categories that summarised Caspi et al.’s [5] longitudinal data. This, while providing a neater data set to demonstrate the usefulness of the S-1 bifactor model, did diverge from the base ordinal data that were gathered from five different time points in adulthood. Furthermore, to facilitate a neat demonstration, we did not use Monte-Carlo simulations that are often used in simulation research of this kind, and while we based the positive skew of psychopathological symptoms on empirical data, it is likely that it differed to the skews of individual symptoms from Caspi et al. [5]. We therefore encourage further research to use human data with a number of neurocognitive and symptom measures within a S-1 bifactor approach.

Recently, there have been calls for CFA structural models of psychopathology to be developed, validated and crosschecked with exploratory factor analytic (EFA) approaches to mitigate the issues such as collapsing specific factors and over extraction [29]. Future S-1 bifactor models may be synergistically developed through the use of both EFA and CFA. Further, even although we advocate for the use of the correlated factors model and the S-1 bifactor model, future research may also continue to explore the traditional bifactor approach, and the possibility of a universal substantive meaning of the *p*-factor. The S-1 bifactor model allows us to examine specific theoretically important variables (e.g., neurocognitive abilities) within a dimensional psychopathology framework [28]. However, the traditional bifactor approach facilitates a useful description and, in the future, possible explanations of psychopathological symptoms at the population level. Therefore, we welcome future research examining a theoretical conceptualisation of *p* built on top of its statistical make up (e.g., [21,25]).

## 6. Conclusions

In this paper, we showed the utility of the S-1 bifactor approach to the study of neurocognitive abilities and psychopathology. We demonstrated the distinct advantages that the S-1 bifactor model has over the traditional bifactor model for examining the potential contribution of neurocognitive abilities towards psychopathology. Specifically, we show how S-1 bifactor models, using neurocognitive abilities as the reference domain for the general factor, allow for the assessment of each individual indicator’s loadings on the neurocognitive ability referenced general factor, and how those factor loadings and the associations between the specific factors, even if unexpected, can inform hypotheses and theoretical understandings. We also suggest that the correlated factors model and the S-1 bifactor model can be used in parallel to explore associations of neurocognitive abilities and psychopathology due to their distinct ability to answer different research questions and facilitate data interpretation through comparison. Lastly, even though we argue for the benefits of the S-1 bifactor model over a traditional bifactor model for the exploration of neurocognitive abilities in psychopathology, we welcome the possibility of the development of a theoretical, substantive conceptualisation of *p* that is useful on the individual and subgroup level [30], and that can be replicated and is falsifiable.

## Figures and Tables

**Table 1:** Loadings and Associations of the Revised Bifactor Model.

Loadings/Associations	Alc	Can	HD	Tob	CD	MDE	GAD	Fear	OCD	Mania	Schiz	SB-IQ~	WISC-IQ~	WAIS-IQ~	TMT~	RVIP~	Ext~Int
																	−0.366 **
*p* (Unstandardised)	0.284 (0.205)	0.311 (0.251)	0.336 (0.258)	0.391 (0.265)	0.387 (0.304)	0.609 (0.477)	0.589 (0.471)	0.472 (0.328)	0.695 (0.361)	0.969 (0.507)	0.804 (0.428)	−0.252 **	−0.129 **	−0.231 **	0.133 **	−0.181 **	
Ext (Unstandardised)	0.546 (0.394)	0.628 (0.508)	0.558 (0.428)	0.383 (0.260)	0.557 (0.437)	-	-	-	-	-	-	0.000	0.054 **	−0.042 **	−0.045 **	−0.026 **	
Int (Unstandardised)	-	-	-	-	-	0.248 (0.194)	0.394 (0.315)	0.334 (0.233)	-	-	-	−0.004	−0.056 **	0.027 *	0.061 **	0.004	

Note. All loadings significant at *p* < 0.01. Significance for associations indicated with * = significant at *p* < 0.05. ** = significant at *p* < 0.01. *~ =* Covariation. Alc = Alcohol. Can = Cannabis. HD = Hard Drugs. CD = Conduct Disorder. MDE = Major Depressive Episode. GAD = Generalized Anxiety Disorder. Fear = Fears and Phobias. OCD = Obsessive Compulsive Disorder. Schiz = Schizophrenia. SB-IQ = Stanford–Binet Intelligence Scale Intelligence Quotient. WISC-IQ = Wechsler Intelligence Scale for Children Intelligence Quotient. WAIS-IQ = Wechsler Adult Intelligence Quotient. TMT = Trail Making Test. RVIP = Rapid Visual Information Processing. Ext = Externalising. Int = Internalising.

**Table 2:** Loadings and Associations of the Correlated Factors Model.

Loadings/Associations	Alc	Can	HD	Tob	CD	MDE	GAD	Fear	OCD	Mania	Schiz	SB-IQ~	WISC-IQ~	WAIS-IQ~	TMT~	RVIP~	Ext~Int	Ext~Tht	Int~Tht
																	0.307 **	0.530 **	0.870 **
Ext (Unstandardised)	0.604 (0.436)	0.679 (0.549)	0.649 (0.498)	0.553 (0.375)	0.687 (0.540)	-	-	-	-	-	-	−0.145 **	−0.031 *	−0.166 **	0.041 *	−0.126 **			
Int (Unstandardised)	-	-	-	-	-	0.685 (0.537)	0.683 (0.546)	0.555 (0.386)	-	-	-	−0.237 **	−0.138 **	−0.206 **	0.144 **	−0.167 **			
Tht (Unstandardised)	-	-	-	-	-	-	-	-	0.695 (0.361)	0.969 (0.507)	0.803 (0.428)	−0.252 **	−0.129 **	−0.231 *	0.133 **	−0.181 **			

Note. All loadings significant at *p* < 0.01. * = significant at *p* < 0.05. ** = significant at *p* < 0.01. ~ = Covariation. Alc = Alcohol. Can = Cannabis. HD = Hard Drugs. CD = Conduct Disorder. MDE = Major Depressive Episode. GAD = Generalized Anxiety Disorder. Fear = Fears and Phobias. OCD = Obsessive Compulsive Disorder. Schiz = Schizophrenia. SB-IQ = Stanford–Binet Intelligence Scale Intelligence Quotient. WISC-IQ = Wechsler Intelligence Scale for Children Intelligence Quotient. WAIS-IQ = Wechsler Adult Intelligence Quotient. TMT = Trail Making Test. RVIP = Rapid Visual Information Processing. Ext = Externalising. Int = Internalising. Tht = Thought Disorder.

**Table 3:** Loadings and Associations of the IQ S-1 Bifactor Model.

Loadings/Associations	SB-IQ	WISC-IQ	WAIS-IQ	Alc	Can	HD	Tob	CD	MDE	GAD	Fear	OCD	Mania	Schiz	Ext~Int	Ext~Tht	Int~Tht
															0.287 **	0.518 **	0.863 **
IQ *g* (Unstandardised)	0.936 (1.00)	0.790 (0.844)	0.826 (0.883)	−0.071 (−0.004)	−0.085 (−0.005)	−0.078 (−0.004)	−0.082 (−0.004)	−0.099 (−0.006)	−0.159 (−0.009)	−0.149 (−0.009)	−0.124 (−0.006)	−0.188 (−0.007)	−0.255 (−0.010)	−0.201 (−0.008)			
Ext (Unstandardised)	-	-	-	0.601 (1.00)	0.674 (1.26)	0.645 (1.14)	0.546 (0.856)	0.679 (1.23)									
Int (Unstandardised)				-	-	-	-	-	0.666 (1.00)	0.668 (1.02)	0.541 (0.722)	-	-	-			
Tht (Unstandardised)				-	-	-	-	-	-	-	-	0.669 (1.00)	0.935 (1.41)	0.778 (1.19)			

Note. All loadings significant at *p* < 0.01. Significance for associations indicated with * = significant at *p* < 0.05. ** = significant at *p* < 0.01. ~ = Covariation. IQ *g* = Intelligence Quotient General Factor. Alc = Alcohol. Can = Cannabis. HD = Hard Drugs. CD = Conduct Disorder. MDE = Major Depressive Episode. GAD = Generalized Anxiety Disorder. Fears = Fears and Phobias. OCD = Obsessive Compulsive Disorder. Schiz = Schizophrenia. SB-IQ = Stanford–Binet Intelligence Scale Intelligence Quotient. WISC-IQ = Wechsler Intelligence Scale for Children Intelligence Quotient. WAIS-IQ = Wechsler Adult Intelligence Quotient. Ext = Externalising. Int = Internalising. Tht = Thought Disorder.

**Table 4:** Loadings and Associations of the Executive Function S-1 Bifactor Model 1.

Loadings/Associations	TMT	RVIP	Alc	Can	HD	Tob	CD	MDE	GAD	Fears	OCD	Mania	Schiz	Ext~Int	Ext~Tht	Int~Tht
														0.273 **	0.512 **	0.854 **
EF *g* (Unstandardised)	0.467 (1.00)	−0.552 (−1.22)	0.046 (0.069)	0.077 (0.130)	0.083 (0.132)	0.174 (0.132)	0.132 (0.217)	0.237 (0.387)	0.223 (0.372)	0.166 (0.241)	0.238 (0.258)	0.329 (0.359)	0.270 (0.300)			
Ext (Unstandardised)	-	-	0.608 (1.00)	0.678 (1.25)	0.645 (1.129)	0.529 (0.818)	0.672 (1.21)									
Int (Unstandardised)			-	-	-	-	-	0.642 (1.00)	0.647 (1.03)	0.531 (0.734)	-	-	-			
Tht (Unstandardised)			-	-	-	-	-	-	-	-	0.653 (1.00)	0.912 (1.41)	0.757 (1.20)			

Note. All loadings significant at *p* < 0.01. Significance for associations indicated with * = significant at *p* < 0.05. ** = significant at *p* < 0.01. * = significant at *p* < 0.05. ~ = Covariation. EF g = Executive Function General Factor. Alc = Alcohol. Cann = Cannabis. HD = Hard Drugs. CD = Conduct Disorder. MDE. Dep = Major Depressive Episode. GAD = Generalized Anxiety Disorder. Fears = Fears and Phobias. OCD = Obsessive Compulsive Disorder. Schiz = Schizophrenia. TMT = Trail Making Test. RVIP = Rapid Visual Information Processing. Ext = Externalising. Int = Internalising. Tht = Thought Disorder.

**Table 5:** Loadings and Associations of the Executive Function S-1 Bifactor Model 2.

Loadings/Associations	TMT-B	RVIP	Alc	Can	HD	Tob	CD	MDE	GAD	Fears	OCD	Mania	Schiz	Ext~Int	Ext~Tht	Int~Tht
														−0.044 *	0.315 **	0.679 **
EF *g* (Unstandardised)	0.849 (1.00)	−0.980 (−1.06)	0.233 (0.140)	0.265 (0.178)	0.282 (0.180)	0.334 (0.189)	0.324 (0.189)	0.517 (0.338)	0.504 (0.336)	0.392 (0.228)	0.593 (0.257)	0.823 (0.359)	0.676 (0.300)			
Ext (Unstandardised)	-	-	0.568 (1.00)	0.640 (1.26)	0.587 (1.10)	0.434 (0.720)	0.599 (1.15)	-	-	-	-	-	-			
Int (Unstandardised)	-	-	-	-	-	-	-	0.437 (1.00)	0.466 (1.09)	0.407 (0.827)	-	-	-			
Tht (Unstandardised)	-	-	-	-	-	-	-	-	-	-	0.362 (1.00)	0.512 (1.43)	0.433 (1.23)			

Note. All loadings significant at *p* < 0.01. Significance for associations indicated with * = significant at *p* < 0.05. ** = significant at *p* < 0.01. ~ = Covariation. EF *g* = Executive Function General Factor. Alc = Alcohol. Can = Cannabis. HD = Hard Drugs. CD = Conduct Disorder. MDE = Major Depressive Episode. GAD = Generalized Anxiety Disorder. Fears = Fears and Phobias. OCD = Obsessive Compulsive Disorder. Schiz = Schizophrenia. TMT = Trail Making Test. RVIP = Rapid Visual Information Processing. Ext = Externalising. Int = Internalising. Tht = Thought Disorder.

## Data Availability

The data and data generation and analysis code will be made available on request to the corresponding author.
